# Predicting adult-age mental health with childhood reading and math disability: do resilience and coping styles matter?

**DOI:** 10.1007/s11881-023-00290-8

**Published:** 2023-10-25

**Authors:** Tuija Aro, Ahmet Bilal Özbek, Minna Torppa

**Affiliations:** 1 Department of Psychology, Centre of Excellence in Learning Dynamics and Intervention Research (InterLearn) University of Jyväskylä, Jyväskylä, Finland; 2 Department of Special Education, Dokuz Eylul University, Izmir, Türkiye; 3 Department of Teacher Education, University of Jyväskylä, Jyväskylä, Finland

**Keywords:** Reading disability, Math disability, Mental health, Resilience, Coping styles

## Abstract

We studied the associations between childhood-identified learning disabilities and adult-age mental health and whether adult-age reading and math skills, coping styles, or resilience influenced the associations. The participants were 159 Finnish adults (60.4% males). Of them, 48 (30%) had a reading disability (RD), 22 (14%) had a math disability (MD), 21 (13%) had RD + MD identified in childhood, and 68 (43%) were population-based controls, matched based on gender, age, and place of residence. At ages 20–40 (*M*_age_ = 29), they reported their mental health, coping styles, and resilience, and their reading and math skills were assessed. The hierarchical regression analyses, predicting mental health with RD, MD, and their interaction while controlling for gender and age, indicated that childhood MD predicted the occurrence of more mental health problems in adulthood, but this was not observed in the case of RD. The RDxMD positive interaction effect reflected better mental health in both the RD and the RD + MD groups than in the MD group. Controlling for adult-age reading and math skills had no effect on the association between MD and mental health outcomes while controlling for resilience and coping styles diminished the impact of MD. Strong resilience without the use of an emotion-oriented coping may thus alleviate the association between MD and mental health. As childhood MD can have long-term associations with mental health problems, these issues need to be addressed in school, at work, and in healthcare. Based on our findings, strengthening effective coping and resilience may be one avenue of support.

Individuals with learning disabilities (LDs) are known to have increased risks of mental health problems in both childhood (Aro et al., 2022; Nelson & Harwood, 2011a, b) and adulthood (Aro et al., 2019; Klassen et al., 2013; Wilson et al., 2009). Furthermore, most studies on the associations between learning disabilities (LDs) and mental health have focused on reading disability (RD) or an unspecified LD. Math disability (MD) has been less explored, and typically, MD and RD co-occurrence (RD + MD) has not been controlled for in RD studies. However, recent findings among children indicate that MD in particular might be related to mental health problems (Aro et al., 2022; Wakeman et al., 2023), but research among adults is scarce. Thus, there is little knowledge about the contributions of MD and RD + MD to adult-age mental health problems, which is surprising because it is known that RD and MD often co-occur (Moll et al., 2014) and persist into adulthood (Eloranta et al., 2018; Maughan et al., 2009; Wilson et al., 2015). Therefore, in this study, we examine the associations between childhood RD and MD and adult-age mental health problems. In addition, we aim to identify the factors that could explain the individual variations in adult-age mental health outcomes among people with LDs. The identification of such factors could provide ways to alleviate the elevated risk of mental health problems.

## RD, MD, RD + MD, and mental health

Research among individuals with RD has reported increased numbers of several types of mental health or behavioral problems. Studies among children have shown increased symptoms of anxiety and depression, more somatic complaints, withdrawal, and aggressive and delinquent behavior (Carroll et al., 2005; Livingston et al., 2018; Mammarella et al., 2016; Novita et al., 2019; Willcutt & Pennington, 2000). Despite fewer studies among adults with RD, they have also reported depression, somatic complaints, social problems, and low self-esteem (e.g., Ghisi et al., 2016; Hellendorn & Ruijssenaars, 2000; Klassen et al., 2013; McNulty, 2003).

Although much less studied, MD has also been associated with childhood problems such as anxiety, depression, eating disorders, somatization, oppositional defiant disorder, and conduct problems (e.g., Auerbach et al., 2008; Torppa et al., 2023; Wakeman et al., 2023; Willcutt et al., 2013). Accordingly, a recent longitudinal study based on health-register data stated that childhood MD was more strongly associated with mental health diagnoses in adolescence and adulthood compared with childhood RD (Eloranta et al., 2021) and that MD in particular was associated with an increased use of antidepressants (Aro et al., 2019).

RD and MD often co-occur in childhood (Joyner & Wagner, 2020; Moll et al., 2014), and in adulthood (Wilson et al., 2015). As people with co-occurring disabilities are expected to experience broader difficulties in learning and receive more negative feedback, they possibly face more mental health problems in comparison to people with a single LD. The empirical evidence for this hypothesis is scarce and contradictory. It has been reported that RD + MD is related to increased internalizing psychopathology in childhood (Willcutt et al., 2013). However, Martínez and Semrud-Clikeman (2004) found no differences in emotional symptoms between adolescents with multiple LDs and those with a single disability. Similarly, in Finnish samples of school-aged children (Aro et al., 2022) and adults (Aro et al., 2023), no major differences were observed between people with a single LD and those with multiple LDs.

Not all studies have reported increased numbers of mental health problems among people with a single LD. Two follow-up studies using clinical samples (children: Strehlow et al., 1992; adults: Schulte-Körne et al., 2003) found that the proportions of psychiatric symptoms among individuals with RD were similar to peers with no RD. Likewise, several non-clinical studies have not observed differences in internalizing problems between students with LD and peers with no LD (e.g., Carroll et al., 2005; Miller et al., 2005; Parhiala et al., 2015; Torppa et al., 2023). These mixed findings signify the need to gain a better understanding of the factors contributing to the variance in mental health outcomes and the factors affecting the association between LD and mental health.

### Individual characteristics alleviating the association between RD, MD, and mental health problems

Although the persistence of both RD and MD has been shown, they do not always continue in adulthood. Studies have indicated that for some individuals with early-identified RD, their reading skills can improve considerably over time (e.g., Psyridou et al., 2020; Torppa et al., 2015). However, there is less knowledge about changes in the case of MD, as well as whether the resolving tendency is associated with mental well-being. Therefore, we want to explore whether adult-age skills would alleviate the association between childhood LDs and adult-age mental health problems.

In their follow-up studies among individuals with LDs, Raskind et al. (1999) and Goldberg et al. (2003) have indicated that success in adulthood is related more to non-cognitive factors (e.g., self-awareness, emotional stability) than to cognitive factors (IQ). More recently, based on findings among individuals with RD, it has been suggested that success in different fields may depend on personality and motivational rather than cognitive factors (Łockiewicz et al., 2014). Despite the apparent relevance of non-cognitive factors for well-being, there is little knowledge about which factors positively influence the life course of individuals with LDs.

Although it is intuitive to suppose that LD itself, and especially its related secondary problems (e.g., poor academic performance, low self-esteem), may be sources of increased mental health problems, it might also be the case that the encountered difficulties can build resilience. Resilience is not a fixed attribute (Rutter, 1990); rather, it can be understood as a process, trait, or outcome. When perceived as a personal trait, it is considered an attribute that helps an individual cope with adversity and achieve adjustment (Connor & Davidson, 2003), and it is related to better mental health (Hu et al., 2015).

Supporting the above stance, the research findings among individuals with LDs have shown that the difficulties can generate resilience-promoting experiences (McNulty, 2003; Nalavany et al., 2011). Accordingly, the view that resilience could alleviate mental health problems among individuals with LDs (Haft et al., 2016) is supported by the findings indicating that increased grit and resilience have been associated with less anxiety and depression among children with RD (Hossain et al., 2022) and that better resilience explains general, self-, and social satisfaction (Stack-Cutler et al., 2015). Thus, it can be hypothesized that after a setback, a low level of resilience may promote an anticipation of failure, which, in turn, can reduce resilience, creating a vicious circle leading to poorer mental health. The other way around, good resilience may increase perseverance and thereby further indorse resilience and mental well-being. However, other studies have shown no differences in resilience between students with RD and control group (Ghisi et al., 2016) or have suggested that RD is associated with lower resilience in adult-age (Kalka & Łockiewicz, 2018). Little is known about individuals with MD; thus, more research is needed, especially on this group.

Another individual characteristic related to well-being and to dealing with hardship in life is coping style or strategy. It refers to individual’s ways of reacting to stressors and dealing with them and has also been associated with mental health (e.g., Steiner et al., 2002). In this study, we target emotion- and avoidance-oriented coping styles as characteristics that have a possible negative association with mental health. The former refers to focusing on emotional reactions instead of seeking to solve the problem. The latter involves avoidance of an unpleasant situation, for instance, by withdrawal and disengagement. Thus far, we know little of the coping styles used by individuals with LDs and even less about the association between their coping styles and mental health. To our knowledge, there are no studies on coping styles among adults with MD or RD + MD. Among adults with RD, avoidance has been observed as a common way of coping (Doikou-Avlidou, 2015; Givon & Court, 2010; Hellendoorn & Ruijssenaars, 2000). Among children and adults with RD, their uses of emotion- and avoidance-oriented coping styles have been found to contribute to increased depression and anxiety (Alexander-Passe, 2006; Bazen et al., 2023; Carroll & Iles, 2006). However, other studies have reported no association between LD or RD and coping styles (Gallegos et al., 2012; Novita et al., 2019).

### Present Study

We examined whether the adult participants with childhood-identified RD, MD, or RD + MD self-reported more mental health problems in adult age than their peers. To understand variations in adult-age mental health problems we examined the associations between childhood-identified LDs and adult-age mental health and analyzed whether adult-age reading and math skills, coping styles, or resilience influenced the associations. Furthermore, to be able to draw conclusions on mental health problems related to childhood LDs (RD or MD or both), we aimed to reduce sample heterogeneity in terms of childhood comorbid problems and included in this study only those participants who did not show major attentional or emotional problems in childhood.

## Method

### Participants

The participants were adult individuals with LDs identified in school-age when they attended the Clinic for Learning Disorders (CLD) and their population-based controls. The CLD offers (neuro)psychological assessment and counseling for children with learning difficulties. It is clinic upheld by the Niilo Mäki Institute (NMI) and Jyväskylä Family Counseling Center. Children with social-emotional difficulties as their primary problem or with global developmental delay are not referred to the center. Before a referral, a child’s learning (dis)ability is assessed by the special education teacher/school psychologist. If the difficulties persist despite individually planned and intensified educational support in school, a multi-professional decision-making team refers the child to the CLD. This multi-tiered framework resembles the Response to Intervention model (Fletcher & Vaughn, 2009). It has been officially implemented in Finland since 2010, but in the area of Jyväskylä, it was already used in the late 1980s when the oldest participants of this study were assessed at the CLD.

The participants were selected from the database of the CLD, based on two criteria: they had clear LDs (i.e., reading and/or math test *z*-score ≤ − 1.5) in childhood and were over 20 years old in 2013—at the start of the study that examined the adult-age outcomes of the CLD former clients. Individuals with emotional and attention problems in childhood (*z*‐score ≤ − 1.0; teacher/guardian ratings) were excluded. Child Behavior Checklist (CBCL) and Teacher Rating Forms (TRF) of the Achenbach System of Empirically Based Assessment (ASEBA; Achenbach & Rescorla, 2001) was used to assess these problems.

The participants voluntarily joined the study and signed the informed consent form. Their parents had also given their informed consent to use the childhood data for research purposes. Ethical approval was obtained from the University of Jyväskylä Ethical Committee, and the institutional consent was provided by the NMI.

Based on the above criteria, 76 individuals were identified as eligible RD participants. Forty-nine individuals (74.2% of the 66 contacted individuals) agreed to participate; one participant with a low childhood IQ (Full-Scale IQ: 60) was dropped from the sample. Thirty-eight individuals with MD were identified, 34 were contacted, and 22 (64.7%) agreed to participate. Forty‐two individuals were identified with RD + MD, of whom 33 were contacted, and 21 (63.6%) agreed to participate. The reasons for failing to reach the other potential participants were as follows: no contact information was found, or the person did not answer, is deceased, or lived abroad. To examine the effect of attrition, we compared those participating and those invited but not participating in the adult-age follow-up within each subgroup. There were no significant differences in the childhood skill measures, FSIQ (Full-Scale IQ) scores, age at the time of the childhood assessment nor in the childhood affective problem symptoms, anxious symptoms, or ADHD symptoms.

A control group was formed from an adult-age sample provided by the Population Register Center of Finland, in which each participant from the clinical dataset was matched with five control participants based on age, gender, and hometown at the age of seven (i.e., at the beginning of compulsory education). All five matched controls were contacted in random order, with the aim to provide one control participant for each participant with LD. For 10 of the participants with RD, 5 with MD, and 7 with RD + MD, none of their matching 5 control participants was reached, or all declined to participate. One potential RD-control participant with a low FSIQ was dropped from the sample, yielding 37 individuals as RD controls, 17 as MD controls, and 14 as RD + MD controls. The final sample comprised 159 adults, of whom 48 (30%) had RD, 22 (14%) had MD, and 21 (13%) had RD + MD diagnosed in childhood, and 68 (43%) were population-based control participants, and there was no available information about the childhood learning status of the control participants.

Gender distribution, educational attainment, and current occupation/situation and childhood measurement scores are reported in Table 1. The mean values of the adult-age FSIQ (WAIS IV; Wechsler, 2008) were as follows: 86.1 (SD = 15.3) for the group with RD, 80.4 (SD = 15.9) for the group with MD, 76.9 (SD = 15.1) for the group with RD + MD, and 99.8 (SD = 15.2) for the control group.

**Table 1:** Study Sample’s Gender Distribution (percentages), Childhood Reading and Math z-Scores, Childhood Emotional and Attentional Problems z-Scores, Educational Attainment and Current Activity (percentages)

	RD	MD			RD + MD			Control				
	n (% of male)		48 (62.50)			22 (50.00)			21 (71.40)	68 (58.80)		
	M (SD)	M (SD)						M (SD)			M (SD)	
Childhood z-Scores ^a)^	Reading		-5.03 (5.26)			-0.02 (1.00)			-4.52 (4.42)			
	Math		-0.66 (0.94)			-2.60 (1.06)			-2.30 (0.81)			
	Anxiety Problems		0.12 (1.13)			0.11 (0.87)			0.03 (0.67)			
	Affective Problems		0.24 (1.17)			0.08 (0.70)			0.29 (1.02)			
	ADHD Symptoms		0.01 (0.80)			-0.47 (1.16)			-0.42 (1.10)			
			% (n)	% (n)			% (n)			% (n)		
Educational Attainment	Comprehensive school		6.25 (3)			4.45 (1)				3.13 (2)		
	Vocational school		68.75 (33)			68.20 (15)			85.71 (18)	32.81 (21)		
	High school		6.25 (3)			4.45 (1)				12.50 (8)		
	University of applied sciences		12.50 (6)			13.64 (3)			9.52 (2)	31.25 (20)		
	University		6.25 (3)			9.09 (2)			4.76 (1)	20.31 (13)		
Current activity	Studying		20.83 (10)			9.09 (2)			9.52 (2)	17.46 (11)		
	Part-time job		6.25 (3)			4.45 (1)			4.76 (1)	11.11 (7)		
	Full-time job		50.00 (24)			63.63 (14)			66.66 (14)	58.73 (37)		
	Unemployed/Job seeking		20.83 (10)			18.18 (4)			9.52 (2)	7.94 (5)		
	Parental leave					4.45 (1)			4.76 (1)	1.58 (1)		
	Sickness leave								4.76 (1)	3.17 (2)		
	Military service		2.08 (1)									

*Note*. RD = reading disability, MD = math disability, RD + MD = reading and math disability. ^a)^ Finnish normative data-based z-scores of the reading test and math test used with the child in childhood assessment and of the scales of the Achenbach System of Empirically Based Assessment (ASEBA; Achenbach & Rescorla, 2001). Higher score indicates less problems

## Measures

### Identifying LDs in childhood

The identification of LDs was based on the tests used at the time when the children were assessed at the clinic. RD, MD, and RD + MD were identified if the children scored 1.5 SDs below the age- or grade-level mean in the reading and/or math test that they completed at the clinic.

**Reading Fluency.** Since most of the Finnish children achieve accurate reading skills in the first grade, after which RD is manifested mainly as dysfluent reading (Aro & Wimmer, 2003), we identified RD based on reading fluency problems. The measures used were text or wordlist reading tests developed and normed locally (Misku-Text, ÄRPS-Wordlist, and Markkinat-Wordlist) or nationwide in Finland (Lukilasse). In Misku-Text, ÄRPS-Wordlist, and Markkinat-Wordlist, the fluency score is based on the time taken to complete the task, and in Lukilasse the score is obtained by counting the correctly read words within 2 min. The Misku-Text and the Markkinat-Wordlist were normed for 8–12-year-old children (NMI, 1985–2004). In the Misku-Text, the child reads aloud a short story, and in the Markkinat-Wordlist, the child reads a list of 13 words as fluently and correctly as possible. The ÄRPS-Wordlist is a word and pseudoword list-reading test, normed for Grades 2 to 4 (NMI, 1985–2004). Lukilasse (Häyrinen et al., 1999) is a normed test battery for Grades 1 to 6. In the Lukilasse–Word Reading Subtest, the child reads aloud a list of words, and Cronbach’s alpha range is 0.94–0.98, depending on the grade (Häyrinen et al., 1999). Psychometric information is not available for other tests.

**Math.** All participants in the MD and the RD + MD groups were assessed in childhood with the RMAT test (Räsänen, 1992), but the math skills of the RD group members were assessed with one of the following: the RMAT test, the Kaufman Assessment Battery for Children (K-ABC; Kaufman & Kaufman, 1983), or Lukilasse (Häyrinen et al., 1999). In the RMAT test (normed for Grades 3 to 5), the child performs as many basic arithmetic operations as possible in 10 min. The score is the number of correct responses. Cronbach’s alpha (0.86) and test-retest reliability have been shown to be good (*r* = .82, 6-month interval; Räsänen, 1992). The K-ABC–Arithmetic Subtest assesses a child’s ability to count, compute, identify numbers, and understand mathematical concepts. Cronbach’s alpha has been at least 0.84 (Matazow et al., 1991). Local norms are available for Grades 2 to 5. The Lukilasse–Arithmetic Subtest consists of basic arithmetic operation tasks, and it is normed for Grades 1 to 6. The Cronbach’s alpha range is 0.55–0.83, depending on the grade (Häyrinen et al., 1999).

### Assessment of academic skills in adulthood

**Reading Fluency.** Reading skills were measured using the pseudoword list-reading task of the test battery on reading and spelling skills for adolescents and adults (Nevala et al., 2006). In the task, the person reads aloud 30 pseudowords as fluently and accurately as possible. The score used in this study was the reference data-based scale scores for the time taken to read the pseudowords (min = 1, max = 9).

**Math.** The RMAT test (Räsänen, 1992) was also used in the adult-age follow-up assessment. The raw score (the number of correct answers) was used in the analyses (min = 0, max = 56).

### Resilience and coping

**Resilience**. The Connor–Davidson Resilience Scale–10 (Campbell-Sills & Stein, 2007) was used to assess resilience. It comprises 10 statements concerning the capacity to cope with adversity (e.g., “I am not easily discouraged by failure”). Participants respond to statements on a 5-point scale, ranging from 1 = “not true at all” to 5 = “true nearly all the time.” The sum score of all items was used. Cronbach’s alpha has been reported to be 0.85 (Campbell-Sills & Stein, 2007) and it was 0.94 in the present sample.

**Coping.** Two scales from the Coping Inventory for Stressful Situations (CISS; Endler & Parker, 1990) were used to evaluate two coping styles: emotion-oriented and avoidance-oriented coping. The items of the emotion-oriented (e.g., “I blame myself for the situations”) and the avoidance-oriented (e.g., “Treat myself to a nice snack”) scales were each evaluated on a 5-point scale, ranging from 0 = “not true at all” to 4 = “true nearly all the time.” Good internal consistencies (Cronbach’s alpha range: 0.82–0.90) have been found (Cosway et al., 2000) and it was 0.90 in the present sample for the emotion-oriented scale and 0.80 for the avoidance-oriented scale.

### Mental health self-reports

**Depression Symptoms.** Depression symptoms were assessed with the Beck Depression Inventory–II (Beck et al., 1996), a 21-item, self-rating questionnaire. Participants respond to statements on a scale ranging from 0 to 3 in the intensity of the symptom, such as self-dislike (e.g., 0 = “I don’t feel disappointed in myself”; 3 = “I hate myself”). The internal consistency estimates of reliability have been good (0.90) (Storch et al., 2004) and Cronbach’s alpha in the present sample was 0.66. The Finnish reference data-based *z*-scores of the sum scores of the scales (Honkalampi et al., 2017) were used in both the BDI and the Clinical Outcomes of Routine Evaluation–Outcome Measure (CORE-OM), with higher scores indicating less symptoms.

**Psychological Distress.** Three domains of the CORE-OM (Evans et al., 2002) were used to assess psychological distress. The CORE-OM includes four domains: Problem Symptoms (12 items, CORE-OM-Problems), Well-Being (4 items, CORE-OM-Well-being), Functioning (12 items, CORE-OM-Functioning), and Risks (6 items; not used in this study). The Problem Symptoms domain taps into psychological health, such as anxiety and depression symptoms, and physical complaints (e.g., “I have felt tense, anxious, or nervous”). In the Well-Being domain, the statements describe self-satisfaction and hopefulness (e.g., “I have felt O.K. about myself”). In the Functioning domain, the statements describe functioning mainly with respect to social encounters (e.g., “I have achieved the things I wanted”). Participants respond to statements on a 3-point scale, ranging from 0 = “not at all” to 2 = “most of the time.” Cronbach’s alpha values of all domains have been reported to vary between 0.77 and 0.97 in the Finnish sample (Honkalampi et al., 2017). The Cronbach’s alpha in the present sample was 0.67 for CORE-OM-Problems, 0.86 for CORE-OM-Well-being, and 0.74 for CORE-OM-Functioning.

## Results

### Descriptives

Table 2 reports the descriptives and the RD, MD, and RD + MD group comparisons. The distributions of the measures resembled normal distributions, and there were no extreme outliers, except for the depression symptoms (BDI) with some kurtosis and skewness, and four outliers at the lower end of the distribution. As the results remained the same after winsorizing the outliers to the end of the distribution, the results are reported with the original BDI variable.

**Table 2:** Descriptive Statistics and Group Comparisons

	RD (N = 48)		MD (N = 22)			RD + MD (N = 21)		Control (N = 68)			ANOVA			
	M	SD	M	SD	M		SD		M	SD		F (3.147-158)	Pairwise comparisons (Scheffe)	
Age (yrs)	26.23	4.75	32.27	4.05	30.57		2.46		29.34	4.72		11.36^***^	RD < MD, RDMD, Control	
Adult-age skills														
Reading (scale score)	3.40	2.74	4.82	2.44	2.24		1.51		4.90	2.16		9.05^***^	RD < Control	
													RDMD < MD, Control	
Math (raw score)	34.39	6.71	26.86	4.83	27.33		5.82		37.56	7.34		21.10^***^	MD < RD, Control	
													RDMD < RD, Control	
Mental health (z-scores)														
CORE-OM-Problems	-0.29	0.97	-1.32	1.69	-0.05		1.56		-0.44	1.14		4.53^**^	MD < RD, RDMD, Control	
CORE-OM-Well-being	0.62	0.35	0.33	0.56	0.62		0.66		0.68	0.36		3.67^*^	MD < Control	
CORE-OM-Functioning	-0.59	1.03	-1.47	1.36	-1.03		1.59		-0.31	1.08		6.09^**^	MD < Control, MD < RD	
Depression symptoms (BDI)	0.00	0.69	-1.24	1.74	-0.38		1.54		0.01	0.75		8.17^***^	MD < RD, RDMD, Control	
Coping strategy (raw scores)														
Emotion-oriented	9.33	4.02	20.19	5.86	16.70		5.72		12.47	5.64		24.55^***^	RD < MD, RDMD, Control	
													Control < RDMD, MD	
Avoidance-oriented	10.83	4.58	17.57	4.25	15.20		3.41		10.76	4.38		18.30^***^	RD < RDMD, MD	
													Control < RDMD, MD	
Resilience (raw scores)	2.99	0.56	3.72	0.52	3.96		0.79		3.41	0.76		11.76^***^	RD < MD, RDMD, Control	

*Note*. *p < .05, **p < .01, ***p < .001. RD = reading disability, MD = math disability, RD + MD = reading and math disability

The ANOVA group comparisons suggested significant differences in all measures. The RD group was, on average, somewhat younger than the other groups, had poorer adult-age reading skills than the control and the MD groups, and reported the lowest resilience. The RD group’s math skills, mental well-being scores, and coping scores were comparable to those of the control group. The MD group had poorer adult-age math scores than the control and the RD groups, had the lowest mental health scores, and reported higher levels of emotion-oriented and avoidance-oriented coping than the control and the RD groups. Their ages, adult-age reading skills, and resilience scores did not differ significantly from those of the control group. The RD + MD group had poorer adult-age reading skills than the MD and the control groups, had poorer adult-age math skills than the control and the RD groups, and reported higher levels of emotion-oriented and avoidance-oriented coping than the control and the RD groups. Their ages, mental health scores, and resilience scores were at levels similar to those of the control group. We also counted the percentages of individuals still showing LD in adult-age. Based on the reading fluency task, 42.6% in the RD group and 57.1% in the RD + MD group still had RD when performance − 1.5 SDs below the normative data mean was used as the cut-off. Based on the math test, 36.4% in the MD group and 38.1% in the RD + MD group still had MD when performance − 1.5 SDs below the normative data’s 5th grade mean was used as the cut-point.

To better understand the severity of the mental health problems in this sample, we counted percentages of individuals reporting rather severe problems using a cut-off score − 1.5 SDs below the normative data’s mean. The percentages were the highest in the MD group. Percentages for the depression symptoms (BDI) were 6.5% in RD, 30.5% in MD, 9.5% in RD + MD, and 7.7% in the control group. Percentages for the CORE-OM-Problems were 12.5% in RD, 40.9% in MD, 9.5% in RD + MD, and 20.6% in the control group. Percentage of individuals scoring below the cut-off in the CORE-OM-Functioning were 20.8% in RD, 45.5% in MD, 28.6% in RD + MD, and 14.7% in the control group. There were no individuals scoring below the cut-off in CORE-OM-Well-being.

### Predicting mental health scores

Table 3 reports the correlations between the variables across the full sample. Age was negatively correlated with CORE-OM-Problems and depression symptoms (BDI) and positively correlated with coping and resilience scores, indicating that the older participants reported poorer mental health, higher resilience, and higher levels of emotion-oriented and avoidance-oriented coping. Adult-age reading fluency was not correlated with any of the other measures, while math skills were negatively correlated with coping and resilience skills. The mental health indicators correlated strongly with each other, negatively with the coping measures, and positively with the resilience measure. The better the participants’ mental health, the less they used emotion-oriented and avoidance-oriented coping, and the higher their resilience scores.

**Table 3:** Correlation Table (Pearson) between Adult-Age Measures

		1	2	3	4	5	6	7	8	9
1.	Age									
2.	Reading skills	0.03								
3.	Math skills	− 0.08	0.16							
4.	CORE-OM-Problems	− 0.18^*^	− 0.13	− 0.08						
5.	CORE-OM-Well-being	− 0.15	0.02	0.08	0.78^***^					
6.	CORE-OM-Functioning	− 0.14	0.13	0.11	0.65^***^	0.76^***^				
7.	Depression symptoms (BDI)	− 0.25^**^	− 0.11	0.10	0.75^***^	0.70^***^	0.62^***^			
8.	Emotion-oriented coping	0.31^***^	0.13	− 0.25^**^	− 0.43^***^	− 0.43^***^	− 0.43^***^	− 0.51^***^		
9.	Avoidance-oriented coping	0.23^**^	0.02	− 0.35^***^	− 0.16^*^	− 0.27^***^	− 0.17^*^	− 0.34^***^	0.54^***^	
10.	Resilience	0.21^*^	0.12	− 0.20^*^	0.30^***^	0.30^***^	0.25^**^	0.18^*^	0.11	0.15

*Note*. *p < .05, **p < .01, ***p < .001

Tables 4, 5 and 6 report hierarchical regression analyses predicting each mental health score. In these models, age and gender were entered as control variables in step 1, and RD, MD, and the RDxMD interaction entered in step 2. Because there were differences in the FSIQs between the groups, we ran all the hierarchical regression analyses also with FSIQ entered at the first step together with age and gender. However, these analyses are not reported because the *β*s for FSIQ were not statistically significant nor did this inclusion change the *β*s of the RD, MD or RDxMD in any of the following models.

**Table 4:** Standardized Estimates, Standard Errors, and R2s of Hierarchical Regression Analyses Predicting Mental Health Scores with Age, Gender, Childhood RD, MD, and RD*MD

	Depression symptoms (N = 149)			CORE-OM Problems (N = 156)			CORE-OM Well-Being (N = 156)			CORE-OM Functioning (N = 156)			
	*β*	B (s.e.)	∆*R*^2^ *F*(*df*_1_;*df*_2_)	*β*	B (s.e.)	∆*R*^2^ *F*(*df*_1_;*df*_2_)	*β*	B (s.e.)	∆*R*^2^ *F*(*df*_1_;*df*_2_)	*β*	B (s.e.)	∆*R*^2^ *F*(*df*_1_;*df*_2_)	
Step 1			0.09^***^ 7.72^***^ (2;149)			0.05^*^ 4.04^*^ (2;156)			0.05^*^ 3.76^*^ (2;156)			0.02 1.70 (2;156)	
Age	− 0.26**	− 0.06 (0.02)		− 0.19^*^	− 0.05 (0.02)		− 0.17^*^	− 0.02 (0.01)		− 0.14	− 0.04 (0.02)		
Gender	− 0.42^*^	− 0.18 (0.18)		− 0.13	− 0.34 (0.21)		− 0.15	− 0.15 (0.07)		0.04	0.11 (0.20)		
Step 2			0.09^***^ 6.75^***^ (5;146)			0.06^*^ 3.57^**^ (5;153)			0.05^*^ 3.22^**^ (5;153)			0.09^**^ 3.92^**^ (5;153)	
Age	− 0.18*	− 0.04 (0.02)		− 0.15	− 0.04 (0.02)		− 0.13	− 0.01 (0.01)		− 0.07	− 0.02 (0.02)		
Gender	− 0.37^*^	− 0.16 (0.17)		− 0.10	− 0.26 (0.20)		− 0.14	− 0.13 (0.07)		0.06	0.14 (0.20)		
RD	− 0.07	− 0.15 (0.21)		0.01	0.02 (0.24)		− 0.12	− 0.11 (0.09)		− 0.13	− 0.33 (0.24)		
MD	− 0.44^***^	-1.10 (0.27)		− 0.26^*^	− 0.74 (0.31)		− 0.31^**^	− 0.31 (0.11)		− 0.40^***^	-1.120 (0.30)		
RDxMD	0.27^*^	0.87 (0.38)		0.30^*^	1.12 (0.45)		0.26^*^	0.34 (0.16)		0.21	0.77 (0.43)		
*R* ^2^			0.19			0.11			0.10			0.11	

*Note*. ***p ≤ .001, **p ≤ .01, *p ≤ .05. RD = reading disability, MD = math disability, RD + MD = reading and math disability

First, the main effects of RD, MD, and their interaction on the mental health scores were examined (Table 4). The regression models predicted in total 10–19% of the mental health variance, and the highest *R*^2^ was found for the depression symptoms. Of the control measures, age and gender were significant only in the depression symptoms model; younger participants reported less depression symptoms, and female participants reported more. Step 2 with RD, MD, and the RDxMD interaction as predictors significantly increased the predicted variance in all mental health scores (5–9%). Of the predictors in step 2, childhood MD was a significant predictor in all models, whereas RD was not significant in any of the models. The RDxMD interaction effect was significant in all models except CORE-OM-Functioning. The significant positive regression estimates in step 2 suggested that with respect to all included scales, having childhood MD predicted poorer mental health in adulthood (Table 2). The RDxMD interaction emerged because the participants with comorbid problems reported better mental health than the MD group participants (Table 2).

We run the simple slopes tests for the mental health outcomes with statistically significant RDxMD interactions effect. For the depression symptoms, the analyses indicated that if the participant did not have MD, RD had no statistically significant effect (*β* = -0.152; *p* = .459). However, if the participant had MD, also RD made a significant contribution to the depression symptoms and the effects was positive (*β* = 0.714; *p* = .029), that is, the standardized BDI score became higher indicating less depression symptoms. The same was true for the CORE-OM-Problems; in case of not having MD, RD did not have a statistically significant effect (*β* = 0.019; *p* = .938), but in case of having MD, RD made a significant positive contribution (*β* = 1.143; *p* = .003). A similar positive effect of RD in case of having MD emerged for the CORE-OM-Well-being, but it only approached significance (*β* = 0.238; *p* = .070). In case of not having MD, the effect of RD was not statistically significant (*β* = -0.106; *p* = .207).

### Do adult-age reading and math skills or coping styles and resilience alleviate the association between childhood LD and mental health?

We examined whether adult-age reading and math skills alleviated the predictive effects of childhood MD on adult-age mental health scores. Reading and math skills were entered as predictors in step 2. Adding adult-age skills did not improve the prediction of mental health indicators and did not change the regression estimates for RD and MD. The results (Table 5) thus indicated that adult-age skills did not modify the impact of childhood LDs on adult-age mental health.

**Table 5:** Standardized Estimates, Standard Errors, and R2s of Hierarchical Regression Analyses Predicting Mental Health Scores with Age, Gender, Childhood RD, MD, RD*MD, and Adult-Age Reading and Math Skills

	Depression symptoms (N = 139)			CORE-OM Problems (N = 146)			CORE-OM Well-Being (N = 146)			CORE-OM Functioning (N = 146)				
	*β*	B (s.e.)	∆*R*^2^ *F*(*df*_1_;*df*_2_)		B (s.e.)	∆*R*^2^ *F*(*df*_1_;*df*_2_)	*β*	B (s.e.)	∆*R*^2^ *F*(*df*_1_;*df*_2_)		*β*	B (s.e.)	∆*R*^2^ *F*(*df*_1_;*df*_2_)	
Step 1			0.08^*^ 5.92^**^ (2;139)			0.04^*^ 3.29^*^ (2;146)			0.04 2.64 (2;146)				0.02 1.14 (2;146)	
Age	− 0.22^**^	− 0.05 (0.02)		− 0.17^*^	− 0.05 (0.02)		− 0.13	− 0.01 (0.01)			− 0.11	− 0.03 (0.02)		
Gender	− 0.19^*^	− 0.44 (0.19)		− 0.14	− 0.37 (0.22)		− 0.14	− 0.13 (0.08)			0.05	0.14 (0.22)		
Step 2			0.02 3.58^**^ (4;139)			0.02 2.43 (4;146)			0.00 1.45 (4;146)				0.02 1.43 (4;146)	
Age	− 0.21^*^	− 0.05 (0.02)		− 0.17^*^	− 0.05 (0.02)		− 0.13	− 0.01 (0.01)			− 0.10	− 0.03 (0.02)		
Gender	− 0.17^*^	− 0.39 (0.19)		− 0.14	− 0.38 (0.22)		− 0.14	− 0.13 (0.08)			0.06	0.14 (0.22)		
Reading skills	− 0.10	− 0.05 (0.04)		− 0.10	− 0.05 (0.04)		0.03	0.01 (0.02)			0.10	0.05 (0.04)		
Math skills	0.09	0.01 (0.01)		− 0.09	− 0.02 (0.01)		0.05	0.00 (0.01)			0.10	0.02 (0.01)		
Step 3			0.09^**^ 4.15^***^ (7;139)			0.06^*^ 2.90^**^ (7;146)			0.05 1.84 (7;146)				0.08^*^ 2.55^*^ (7;136)	
Age	− 0.15	− 0.04 (0.04)		− 0.12	− 0.03 (0.02)		− 0.10	− 0.01 (0.01)			− 0.05	− 0.01 (0.02)		
Gender	− 0.17^*^	**− .3**9 (0.01)		− 0.13	− 0.34 (0.22)		− 0.14	− 0.13 (0.08)			0.05	0.14 (0.21)		
Reading skills	− 0.10	− 0.04 (0.02)		− 0.05	0.03 (0.05)		0.05	0.01 (0.02)			0.10	0.05 (0.05)		
Math skills	0.08	− 0.01(0.01)		− 0.19	− 0.03 (0.02)		− 0.05	0.00 (0.01)			− 0.07	− 0.01 (0.02)		
RD	− 0.10	− 0.24 (0.24)		− 0.02	− 0.06 (0.28)		− 0.10	− 0.09 (0.10)			− 0.10	− 0.26 (0.27)		
MD	− 0.47^**^	-1.17 (0.31)		− 0.36^**^	-1.03 (0.36)		− 0.33^*^	− 0.33 (0.12)			− 0.43^***^	-1.21 (0.35)		
RDxMD	− 0.26^*^	0.82 (0.40)		0.31^*^	1.14 (0.47)		0.27^*^	0.36 (0.17)			0.24	0.86 (0.45)		
*R* ^2^			0.18			0.13			0.09				0.11	

*Note*. ***p ≤ .001, **p ≤ .01, *p ≤ .05. RD = reading disability, MD = math disability, RD + MD = reading and math disability

**Table 6:** Standardized Estimates, Standard Errors, and R2s of Hierarchical Regression Analyses Predicting Mental Health Scores with Age, Gender, Childhood RD, MD, RD*MD, Coping Strategies, and Resilience

	Depression symptoms (N = 149)			CORE-OM Problems (N = 156)			CORE-OM Well-Being (N = 156)			CORE-OM Functioning (N = 156)			
	*β*	B (s.e.)	∆*R*^2^ *F*(*df*_1_;*df*_2_)	*β*	B (s.e.)	∆*R*^2^ *F*(*df.*_1_;*df*_2_)	*β*	B (s.e.).	∆*R*^2^ *F*(*df*_1_;*df*_2_)	*β*	B (s.e.)	∆*R*^2^ *F*(*df*_1_;*df*_2_)	
Step 1			0.10^***^ 7.86^***^ (2;149)			0.05^*^ 4.17^*^ (2;156)			0.05^*^ 3.89^*^ (2;156)			0.03 1.94 (2;156)	
Age	− 0.27^***^	− 0.03 (0.02)		− 0.20^*^	− 0.05 (0.02)		0.18^*^	− 0.02 (0.01)		− 0.15	− 0.04 (0.02)		
Gender	− 0.18^*^	− 0.41 (0.18)		− 0.12	− 0.32 (0.21)		0.15	− 0.13 (0.07)		0.05	0.12 (0.20)		
Step 2			0.26^***^ 16.01 (5;149)			0.28^***^ 14.89^***^ (5;156)			0.27^***^ 14.37^***^ (5;156)			0.28^***^ 1313^***^ (5;156)	
Age	− 0.17^*^	− 0.04 (0.02)		− 0.15^*^	− 0.04 (0.02)		0.11	− 0.10 (0.01)		− 0.06	− 0.02 (0.02)		
Gender	− 0.08	− 0.19 (0.16)		− 0.04	− 0.12 (0.18)		0.05	− 0.04 (0.06)		0.14^*^	0.37 (0.18)		
Resilience	0.28^***^	0.41 (0.10)		0.37^***^	0.63 (0.12)		. 0.37^***^	0.22 (0.04)		0.32^***^	0.53 (0.12)		
Coping strategy: Emotion-oriented	− 0.43^***^	− 0.08 (0.02)		− 0.47^***^	− 0.10 (0.02)		0.38^***^	− 0.03 (0.01)		− 0.49^***^	− 0.10 (0.02)		
Avoidance-oriented	− 0.09	− 0.02 (0.02)		0.08	0.02 (0.02)		0.08	− 0.01 (0.01)		0.03	0.01 (0.02)		
Step 3			0.04^*^ 11.58^***^ (8;149)			0.03 10.27^***^ (8;156)			0.02 9.56^***^ (8;156)			0.06^**^ 10.74^***^ (8;156)	
Age	− 0.16^*^	− 0.04 (0.02)		− 0.13	− 0.03 (0.02)		0.11	− 0.10 (0.01)		− 0.06	− 0.01 (0.02)		
Gender	− 0.09	− 0.21 (0.16)		− 0.03	− 0.08 (0.18)		0.05	− 0.05 (0.02)		0.11	0.29 (0.18)		
Resilience	0.31^***^	0.46 (0.11)		0.36^***^	0.61 (0.13)		. 0.38^***^	0.23 (0.05)		0.36^***^	0.61 (0.12)		
Coping strategy: Emotion-oriented	− 0.37^***^	− 0.07 (0.02)		− 0.44^***^	− 0.09 (0.02)		0.36^***^	− 0.03 (0.01)		− 0.42^***^	− 0.08 (0.02)		
Avoidance-oriented	− 0.01	− 0.00 (0.02)		0.10	− 0.03 (0.02)		0.03	0.01 (0.01)		0.14	0.04 (0.02)		
RD	− 0.06	− 0.13 (0.19)		0.01	0.02 (0.22)		− 0.09	− 0.08 (0.08)		− 0.13	− 0.32 (0.02)		
MD	− 0.33^**^	− 0.84 (0.28)		− 0.18	− 0.52 (0.32)		− 0.20	− 0.20 (0.11)		− 0.35^***^	-1.00 (0.30)		
RDxMD	0.21	0.68 (0.35)		0.21^*^	0.83 (0.40)		0.15	0.20 (0.14)		0.13	0.49 (0.39)		
Step 4			0.11^***^			0.05			0.06			0.04	
			9.86 ^***^			6.95^***^			6.64^***^			6.82^***^	
			(14;149)			(14;156)			(14;156)			(14;156)	
Age	− 0.15	− 0.03 (0.02)		− 0.10	− 0.03 (0.02)		− 0.08	− 0.01 (0.01)		− 0.05	− 0.01 (0.02)		
Gender	0.10	− 0.23 (0.15)		− 0.03	− 0.07 (0.18)		− 0.06	− 0.05 (0.06)		0.11	0.27 (0.18)		
Resilience	0.06	0.09 (0.13)		− 0.23^*^	0.40 (0.16)		0.20^*^	0.12 (0.06)		0.23^*^	0.38 (0.16)		
Coping strategy: Emotion-oriented	− 0.28^*^	− 0.05 (0.02)		− 0.31^*^	− 0.06 (0.03)		− 0.33^**^	− 0.02 (0.01)		− 0.37^**^	− 0.07 (0.02)		
Avoidance-oriented	0.02	0.00 (0.03)		0.01	0.00 (0.03)		0.00	0.00 (0.01)		0.19	0.05 (0.03)		
RD	− 0.74	-1.66 (0.94)		− 0.16	− 0.40 (1.16)		− 0.49	− 0.44 (0.41)		− 0.29	− 0.72 (1.13)		
MD	-1.14^*^	-2.92 (1.45)		-1.10	-3.21 (1.75)		-1.07	-1.09 (0.62)		− 0.94	-2.66 (1.71)		
RDxMD	− 0.04	− 0.13 (0.51)		0.13	0.51 (0.62)		0.01	0.01 (0.22)		0.08	0.31 (0.61)		
RDxResilience	0.60	0.39 (0.24)		0.10	0.07 (0.29)		0.35	0.09 (0.10)		0.28	0.20 (0.29)		
RDxEmotion-oriented	0.28	0.05 (0.03)		0.02	0.00 (0.04)		0.15	0.01 (0.01)		0.12	0.02 (0.04)		
RDxAvoidance-oriented	− 0.08	− 0.01 (0.04)		0.09	0.02 (0.05)		0.04	− 0.00 (0.16)		− 0.23	− 0.04 (0.04)		
MDxResilience	1.33^**^	0.86 (0.28)		1.00^*^	0.75 (0.34)		1.19^**^	0.31 (0.12)		0.67	0.48 (0.33)		
MDxEmotion-oriented	− 0.46	− 0.06 (0.04)		− 0.36	− 0.05 (0.04)		0.08	− 0.00 (0.02)		− 0.39	− 0.04 (0.04)		
MDxAvoidance-oriented	0.00	0.00 (0.04)		0.34	0.06 (0.05)		− 0.18	− 0.01 (0.02)		0.21	0.04 (0.05)		
*R* ^2^			0.51			0.41			0.40			0.40	

*Note*. ***p ≤ .001, **p ≤ .01, *p ≤ .05. RD = reading disability, MD = math disability, RD + MD = reading and math disability

Next, we investigated whether controlling for resilience or coping styles would change the predictive effects of childhood LDs on adult-age mental health scores. The results (Table 6) showed that the model where resilience and coping scores were entered in step 2, predicted 26–28% of the mental health variance. The better the participants’ mental health, the higher the resilience scores and the lower the emotion-oriented coping scores. The estimate for avoidance-oriented coping was not significant. After the inclusion of RD, MD, and their interaction in step 3, 34–40% of the variance in the mental health outcomes was predicted by the models. In comparison to the models (Table 4) without resilience and coping scores, the estimates for MD and RDxMD decreased to some extent. For the depression symptoms, the standardized beta for MD decreased from − 0.44 to -0.33 but remained significant. Similarly, in the CORE-OM-Functioning model, there was a decrease in the estimate for MD (from − 0.40 to -0.35), but the estimate remained significant. For the CORE-OM-Problems and the CORE-OM-Well-being models, the changes in the regression estimates (from − 0.26 to -0.18 and from 0.30 to 0.21, respectively) also meant that the estimates for MD became insignificant. The estimates for RDxMD became insignificant after the inclusion of resilience and coping measures, except for the CORE-OM-Problems model. These findings indicated that resilience and not using emotion-oriented coping somewhat alleviated the effects of RD, MD, and their comorbidity on mental health outcomes.

To further test the possibility that the coping style and resilience could act as protective factors for mental health, we conducted additional analyses, where we added all interactions between childhood RD/MD and coping/resilience measures (MDxresilience, MDxavoidance-oriented, MDxemotion-oriented, RDxresilience, RDxavoidance-oriented, RDxemotion-oriented) on an extra step 4. The only significant interaction in step 4 was the one for MDxresilience in the models for depression symptoms, CORE-OM-Problems, and CORE-OM-Well-being. Regarding the model for CORE-OM-Functioning, none of the interactions was significant. This interaction suggests a differential association between having MD (i.e., belonging to either MD or RD + MD group) and mental health on the varying levels of resilience.

To gain a better understanding of the interaction, we compared the correlations between the mental health indicators and resilience among the participants with MD (i.e., MD or RD + MD) and among the other participants. The results showed a significant and positive correlation between resilience and depression symptoms among the participants with MD (*r* = .66^***^), suggesting that the participants belonging to MD or RD + MD groups and having higher resilience scores also had less depression symptoms, whereas lower correlations were found among the other participants (*r* = .05 among controls; *r* = .43^**^ in RD group). Likewise, among the participants with MD, resilience and CORE-OM-Problems showed a significant and positive correlation (*r* = .67^***^), suggesting that the participants belonging to MD or RD + MD and with higher resilience scores also had better psychological health, whereas a weaker association was found among the other participants (*r* = .25^*^ among controls; *r* = .27^*^ in RD group). Similarly, among the participants with MD, resilience and CORE-OM-Well-being showed a significant and positive correlation (*r* = .66^***^), suggesting that the participants belonging to MD or RD + MD and having higher resilience scores also had better self-satisfaction and hopefulness, whereas somewhat weaker association was found among the other participants (*r* = .24^*^ among controls; *r* = .38^**^ in RD group).

## Discussion

In this study, we examined whether the participants with childhood RD, MD, and RD + MD and the control participants would differ in terms of adult-age mental health. We also tested whether considering adult-age reading and math skills, resilience, or coping styles would change the association between LDs and mental health. We obtained the following results. First, childhood MD was associated with poorer adult-age mental health, but such an association was not observed in childhood RD. Likewise, childhood RD + MD comorbidity was not associated with mental health. Childhood LD, age, and gender explained 11–19% of the mental health variance. Second, adult-age skill levels did not change the predictive effect of childhood LD on adult-age mental health, whereas lower levels of emotion-oriented coping and higher resilience alleviated the association to some extent. The mental health indicators were predicted by the MDxresilience interaction, suggesting resilience as a particularly important factor for mental health in the MD group.

Contrary to previous findings on poor mental well-being among individuals with RD (e.g., Ghisi et al., 2016; Livingston et al., 2018; McNulty, 2003), our first main finding was that childhood RD was not associated with any of the mental health indicators in this study. Similar findings have been observed in another Finnish sample (Parhiala et al., 2015; Torppa et al., 2023). This may be related to the rapid reading acquisition among Finnish speaking children (Aro & Wimmer, 2003), because when children quickly proceed to learning by silent reading, there may be less visible points of comparison to peers than when reading aloud. Another reason may be linked to the Finnish educational system that provides early support in schools from highly educated teachers and special education teachers, without requiring LD-diagnosis. However, to gain a proper understanding of the mechanisms leading to positive mental health outcomes among people with RD, we need more research using, for instance, interviews or observational methods in schools. Cross-linguistic and cross-cultural studies would be useful as well.

The MD group had more mental health problems than the other groups, in line with the results of some recent studies among children (Aro et al., 2022; Wakeman et al., 2023) and adults (Aro et al., 2019). The MD group’s scores were the lowest in all included measures tapping into psychological illness from a rather broad perspective, as the items covered such issues as self-satisfaction, hopefulness, and physical complaints in addition to depression and anxiety symptoms. The MD group also reported using more emotion-oriented coping strategies, suggesting this group’s tendency to focus on their emotional reactions instead of trying to solve the problem itself. As previous research on the associations between LD and mental health has mainly ignored MD, our study adds to the existing literature by indicating the relevance of childhood MD for mental well-being later in life.

Together with previous findings (Aro et al., 2019, 2022; Wakeman et al., 2023), our results pose the question of why MD would increase the risk of mental health problems. It can be speculated that MD-related cognitive deficits further complicate learning and coping with challenges caused by MD. For instance, executive function deficits have been associated with math (e.g., Cragg et al., 2017; Willcutt et al., 2013), and they are also known to be relevant for emotion regulation (e.g., Hendricks & Buchanan, 2016). Additionally, compared with reading, math is a cumulative subject, where new content is based on the mastery of earlier contents; therefore, students with MD may be repeatedly faced with their difficulties. Furthermore, it can be supposed that childhood RD may have been more easily recognized than MD by the teachers, and thus less support may have been offered to those with MD. From a broader perspective, the societal significance and values ascribed to math, as well as the attributions attached to MD, may influence an individual’s self-perception and the perceptions of others. However, to understand the processes and mechanisms linking MD and mental health, the association needs to be confirmed in other samples, and its development should be examined longitudinally, targeting a broader mental health profile and supportive factors. Such research should aim to comprehend the meaning of MD in the life course to find ways to support individuals at different developmental stages. Future studies should not ignore comorbid conditions, such as attention and language disorders—or somatic complaints, which have been shown to co-occur with LD (Aro et al., 2023) and which may also be relevant to consider due to their possible association with mental well-being.

Interestingly, RD and MD comorbidity had no added negative effects on mental health as the RD + MD group did not report mental health problems to the same extent as the MD group. This finding contradicts some of the results reported in studies among children (e.g., Willcutt et al., 2013). However, it is consistent with the findings of earlier Finnish studies in which children with RD + MD (Aro et al., 2022) or adults with RD + MD (Aro et al., 2019, 2023) did not show more problems than the single deficit groups.

Our second main finding was that adult-age basic academic skills did not alleviate the predictive effects of RD and MD on adult-age mental health. Previous studies have reported that learning difficulties, at least in reading, can be resolved over time (Psyridou et al., 2020; Torppa et al., 2015). We expected that this positive development might also be reflected in better mental health because resolving difficulties might mean less struggles in education or at work. However, this was not the case in this sample. In contrast, resilience and coping styles had an impact on the association between LD and mental health. The participants who reported higher levels of resilience indicated better mental health, and controlling for resilience diminished the predictive effect of MD on adult-age mental health. Furthermore, the interaction between resilience and having MD (i.e., belonging either to MD or RD + MD group) was a significant predictor of mental health indicators, and a closer examination of the associations showed that resilience seemed to be particularly important in the participants with MD with respect to mental health. These findings support the stance that in the case of having MD, resilience may protect individuals from psychological distress or help offset the factors that increase the risk of experiencing mental health problems, such as the low general self-esteem reported in other samples (e.g., Torppa et al., 2023). These findings imply that in addition to learning support, individuals with LDs should be provided with opportunities to build their resilience skills, such as being proactive and asking for help. Nonetheless, it is noteworthy that the association between resilience and mental health may not be straightforward because, in fact, the lowest resilience score was reported in the RD sample. Thus, our assumption that LD could generate resilience-promoting experiences (McNulty, 2003; Nalavany et al., 2011) and alleviate mental health problems was supported only in the case of having MD. This calls for studies on the academic, cognitive, and psychological developmental processes to gain a better understanding of what experiences build resilience and for whom—and why individuals with RD report less resilience despite indicating less mental health problems.

In addition to resilience, both avoidance-oriented and emotion-oriented coping were associated with poorer mental health, and in particular, groups with MD reported higher levels of these less functional coping styles. The hierarchical regression analyses indicated that emotion-oriented coping significantly contributed to adult-age mental well-being, which confirms the results of previous studies (Alexander-Passe, 2006; Bazen et al., 2023; Carroll & Iles, 2006). It is plausible that individuals experiencing more mental problems are also prone to react emotionally—that is, emotion-oriented coping could be an indicator of their mental stress. However, it is also possible that an emotion-oriented coping strategy mediates between stressful experiences caused by LD and mental health symptoms. The latter case would imply that students with LD should be provided with interventions that aim to support mental health by reinforcing task-oriented coping strategies and minimizing emotion-oriented coping mechanisms. This view is supported by the positive effects reported in studies integrating diverse socio-emotional support (e.g., coping skills, resilience, self-efficacy) and reading instruction or targeting socio-emotional well-being of children with reading difficulties (Aro et al., 2018; Boyes et al., 2021; Frith et al., 2013). However, interventions targeting socio-emotional well-being among children with math difficulties are fewer.

Overall, the present findings emphasize that focusing on academic skills may not suffice when aiming to understand the association between skills and mental health. Instead, we should search for a deeper understanding of the meanings attached to difficulties and consider the personal life history and environmental factors that shape individual coping styles, resilience, and other characteristics over the years.

### Limitations

Some limitations in the present study should be considered when interpreting the results. First, the sample size was small, and replications are needed. However, a large register-based study conducted in Finland reported similar findings indicating that math disability (MD) in particular was associated with antidepressant use (Aro et al., 2019). Due to small sample, we did not have statistical power to identify smaller effects or compute three-way interactions, that is RDxMDxModerator interactions. The voluntary participation caused a bias toward individuals who were willing and able to participate. Although our result indicating the relevance of MD for mental health is consistent with those detected in health register-based studies without attrition (Aro et al., 2019), it should be noted that some register-based studies in Finland suggest that the RD group also has more mental health problems than the control group (Aro et al., 2023). Second, the adult-age assessments were conducted at different ages, ranging from 20 to 40 years. Although we did control for age, future studies with larger samples from different ages should examine how the associations vary among age groups. Third, we used diagnostic tools (BDI and CORE-OM) as continuous measures, and not many of our participants had severe mental health problems. However, the percentage of individuals with problems in a clinical range was large among the MD group. It is possible that different results concerning the moderators would have emerged if mental health problems were diagnosed or more severe among higher percentage of the sample. Accordingly, other types of mental health indicators could have provided different overall insights. Fourth, we did not include individuals with emotional or attentional problems in childhood in the follow-up data collection. This sampling approach enabled us to minimize sample heterogeneity and to focus on purer LDs (RD or MD or both) diminishing the effects of comorbid childhood problems on the results. This allows us to draw clearer conclusions on the association between childhood LDs and adult-age well-being. Thus, our findings should also be interpreted as reflecting outcomes of pure LDs with no comorbid problems. Fifth, the adult-age reading and math skill assessments were based on basic fluency tasks. It is possible that a different picture would have emerged if more functional skills, such as reading comprehension or problem-solving tasks, were evaluated. Finally, the only alleviating factors that we were able to include in this study were coping style and resilience assessed via self-reports at the same time as the mental health outcomes. Future studies should examine these and other factors that could influence the development of the associations between learning and mental well-being, such as environmental or social-interactional factors.

### Conclusions and implications

It can be concluded that childhood MD poses a higher risk of adult-age mental health problems than RD, and RD + MD comorbidity does not seem to increase the risk. Thus, focusing research efforts on RD alone is not sufficient to provide an understanding of mental health problems among individuals with childhood LD; especially, more research efforts should be invested in MD. However, there is no one-to-one association between LD and mental health problems. A clearer understanding of the variations in mental health outcomes requires research on psychological processes, experiences, and the meanings attached by an individual to the difficulty; longitudinal data and qualitative or mixed-method approaches would possibly provide more profound comprehension than data based on questionnaires. A broader perspective, including diverse comorbidities and environmental aspects in addition to personal and socio-emotional factors, is needed as well (e.g., Bazen et al., 2023).

The present findings underscore the need to prevent adult-age mental health problems, especially among people with childhood MD. There are likely multiple avenues to achieve this goal. Based on our results, individuals with MD should be offered opportunities to build their resilience and belief in their self-efficacy to conquer difficulties and bounce back after disappointments. Likewise, helping them acquire better emotion-regulation skills, learn task-oriented coping strategies, and find a self-compassionate stance would probably enable them to avoid unnecessary shame and self-blame. These should be potential options when we aim to identify skills that can alleviate psychological distress and depression symptoms and promote support-seeking among individuals with LDs.
